# Biomarkers for predicting bladder cancer therapy response

**DOI:** 10.32604/or.2024.055155

**Published:** 2025-02-28

**Authors:** IOANA MARIA MIHAI, GANG WANG

**Affiliations:** 1 Department of Pathology and Laboratory Medicine, British Columbia Cancer Vancouver Centre, Vancouver, BC V5Z 4E6, Canada; 2 Department of Pathology and Laboratory Medicine, University of British Columbia, Vancouver, BC V6T 2B5, Canada

**Keywords:** Urine biomarkers, Tissue biomarkers, Blood biomarkers, Precision medicine, Genetic and epigenetic indicators

## Abstract

The advent of precision medicine has underscored the importance of biomarkers in predicting therapy response for bladder cancer, a malignancy marked by considerable heterogeneity. This review critically examines the current landscape of biomarkers to forecast treatment outcomes in bladder cancer patients. We explore a range of biomarkers, including genetic, epigenetic, proteomic, and transcriptomic indicators, from multiple sample sources, including urine, tumor tissue and blood, assessing their efficacy in predicting responses to chemotherapy, immunotherapy, and targeted therapies. Despite promising developments, the translation of these biomarkers into clinical practice faces significant challenges, such as variability in biomarker performance, the necessity for large-scale validation studies, and the integration of biomarker testing into routine clinical workflows. We also highlight the need for standardized methodologies and robust assays to ensure consistency and reliability. Future directions point towards longitudinal studies and the development of combination biomarker panels to enhance predictive accuracy. This review emphasizes the transformative potential of predictive biomarkers in improving patient outcomes and advocates for continued collaborative efforts to overcome existing barriers in this rapidly evolving field.

## Introduction

Bladder cancer (BC) therapy has made considerable progress after years of little advancement in treatment options. BC treatment is complex, and outcomes vary. Traditional management of non-muscle invasive BC (NMIBC) includes transurethral resection followed by intravesical chemotherapy or immunotherapy with Bacillus Calmette-Guérin (BCG). The standard of care for muscle-invasive BC (MIBC) is chemotherapy, partial cystectomy, or radical cystectomy (RC) with pelvic lymph node dissection [1]. Recently, the immune checkpoint inhibitor (ICI) avelumab has been recommended as a first-line maintenance treatment for patients with advanced BC who have not progressed after chemotherapy, and it is offering a promising option for extending survival for this patient group [2,3]. Treatment options for patients with metastatic BC primarily include platinum-based chemotherapy, with cisplatin-containing regimens such as ddMVAC (methotrexate, vinblastine, doxorubicin, cisplatin) and GC (gemcitabine, cisplatin) being the most effective. Patients with metastatic BC, who are not eligible for cisplatin-based chemotherapy, can receive immunotherapy with ICIs like pembrolizumab or atezolizumab for PD-L1 positive patients, and targeted therapy like erdafitinib for FGFR2/3 alterations post-platinum chemotherapy [4–6]. The introduction of antibody-drug conjugates enfortumab vedotin and sacituzumab govitecan, have established new possibilities for locally advanced or metastatic cases, though with the need for careful monitoring of toxicity [7–10]. Recent advances have also emphasized the importance of combination therapies, including the use of immunotherapy and targeted therapy, which may eventually replace platinum-based chemotherapy as the frontline standard of care [10]. Metastasis-directed radiation therapy is another modality explored for its potential to improve oncologic outcomes and quality of life in metastatic BC patients post-RC, with a subset of patients experiencing extended survival [11]. In a review published by Longo et al., the oligometastatic BC patients who were treated with metastasis-directed therapies (metastasectomy and metastasis-directed radiation), had a median overall survival (OS) ranged from 14.9 to 51.0 months and median progression-free survival (PFS) from 2.9 to 10.1 months [11].

Epigenetic therapy in BC represents a promising frontier, targeting reversible alterations in gene expression without changing the DNA sequence itself. This approach is particularly relevant given the prevalence of epigenetic changes in BC, which include DNA methylation and histone modification, among others. The interest in epigenetic therapy stems from its potential to overcome resistance to traditional treatments and improve outcomes for patients with advanced or metastatic BC [12–14]. Clinical trials have explored various epigenetic inhibitors, such as DNA methyltransferase (DNMT) inhibitors and histone deacetylase (HDAC) inhibitors, often in combination with other treatments like chemotherapy or immunotherapy. However, despite the identification of 25 clinical trials investigating epigenetic therapy in BC, Phase 3 trials are yet to be conducted, as this area of research is still in its preliminary stages [12].

Around 30% to 50% of NMIBC patients show no improvement after intravesical BCG therapy and about half of MIBC patients do not respond to neoadjuvant chemotherapy (NAC), losing the chance to benefit from curative RC [15,16]. This inability to predict treatment response has hindered better outcomes for BC patients. Current research on developing new biomarkers for the early detection and prognosis of BC is focused on identifying non-invasive, cost-effective, and accurate methods to improve patient outcomes. Despite the high morbidity and mortality rates associated with BC, traditional detection methods like cystoscopy and cytology face challenges in sensitivity and cost, highlighting the need for better biomarkers for early diagnosis and recurrence monitoring [17]. Recent advances in sequencing technologies have increased our understanding of BC biology and treatment mechanisms; however, biomarkers that predict therapy response for both NMIBC and MIBC are not yet available [18].

Recent studies in BC have found genetic alterations and molecular subtypes that facilitate personalized therapies and biomarker development for treatment prediction [19]. Advanced DNA and RNA profiling technologies present a viable strategy to enhance personalized neoadjuvant therapy and improve patient outcomes [15].

Urine-based liquid biopsy is a new non-invasive method utilized for screening and diagnosis of BC, which uses a wide array of biomarkers including: tumor DNAs (tDNA), proteins, microbiome, tumor RNAs, long non-coding RNAs (lncRNAs), transfer RNA-derived fragments, messenger RNAs (mRNA), microRNAs, circular RNAs, exosomes, and extrachromosomal circular [20]. The exploration of gene mutations, epigenetic modifications, and non-coding RNA (ncRNA) molecules through liquid biopsy has also been highlighted, emphasizing the potential of these biomarkers in the diagnosis and monitoring of BC [21].

Given the complex treatment management of BC patients, biomarkers can prove to be essential in this context, enhancing therapeutic personalization by predicting tumor response to specific treatments, monitoring therapy effectiveness, reducing treatment-related toxicity, providing prognostic insights, and facilitating early detection and surveillance, thereby improving patient outcomes [22–26]. This article intends to provide a complex overview of urine, tissue and blood-based markers that have been assessed for their ability to predict treatment response in BC, as such, this review provides a synthesis of relevant research articles, containing data from a non-systematic review of the literature (Table 1).

**Table 1:** Predictive biomarkers for therapeutic response in patients with bladder cancer known to date

Therapy	Urine based biomarkers/tests	Tissue based biomarkers	Serological biomarkers
BCG therapy	- Urine cytology - UroVysion FISH - ImmunoCyt - CyPRIT	- Molecular subtype classification *- TERT* and *ARID1A* mutations - Epigenetic changes	- CTC
Chemotherapy	- Oncuria - ctDNA	- Mutations in DDR genes - High levels of nuclear CyclinD1 expression - Molecular subtype classification	- CTC - ctDNA
Immunotherapy	- Oncuria - ctDNA	- PD-L1 expression - Mutations in DDR genes - TMB - MSI - TME (CAFs) - TIME (CD8^+^ T cells, TILs)	- Systemic inflammation (NLR; LDH, CRP levels) - ctDNA
Radiotherapy		- Ki-67 expression - Mutations in DDR genes	

Note: **BCG**: Bacillus Calmette-Guérin; **CRP**: C-Reactive Protein; **ctDNA**: Circulating Tumor DNA; **CTCs**: Circulating Tumor Cells; **CTR1**: Copper Transporter 1; **CyPRIT**: Cytokine Panel for Response to Intravesical Therapy; **DDR**: DNA Damage Response; **LDH**: Lactate Dehydrogenase; **MSI**: Microsatellite Instability; **NLR**: Neutrophil-to-Lymphocyte Ratio; **PD-L1**: Programmed Death-Ligand 1; **TILs**: Tumor-Infiltrating Lymphocytes; **TIME**: Tumor Immune Microenvironment; **TMB**: Tumor Mutational Burden; **UroVysion FISH**: UroVysion Fluorescence *in situ* Hybridization.

## Urine Biomarkers

**Figure 1:**
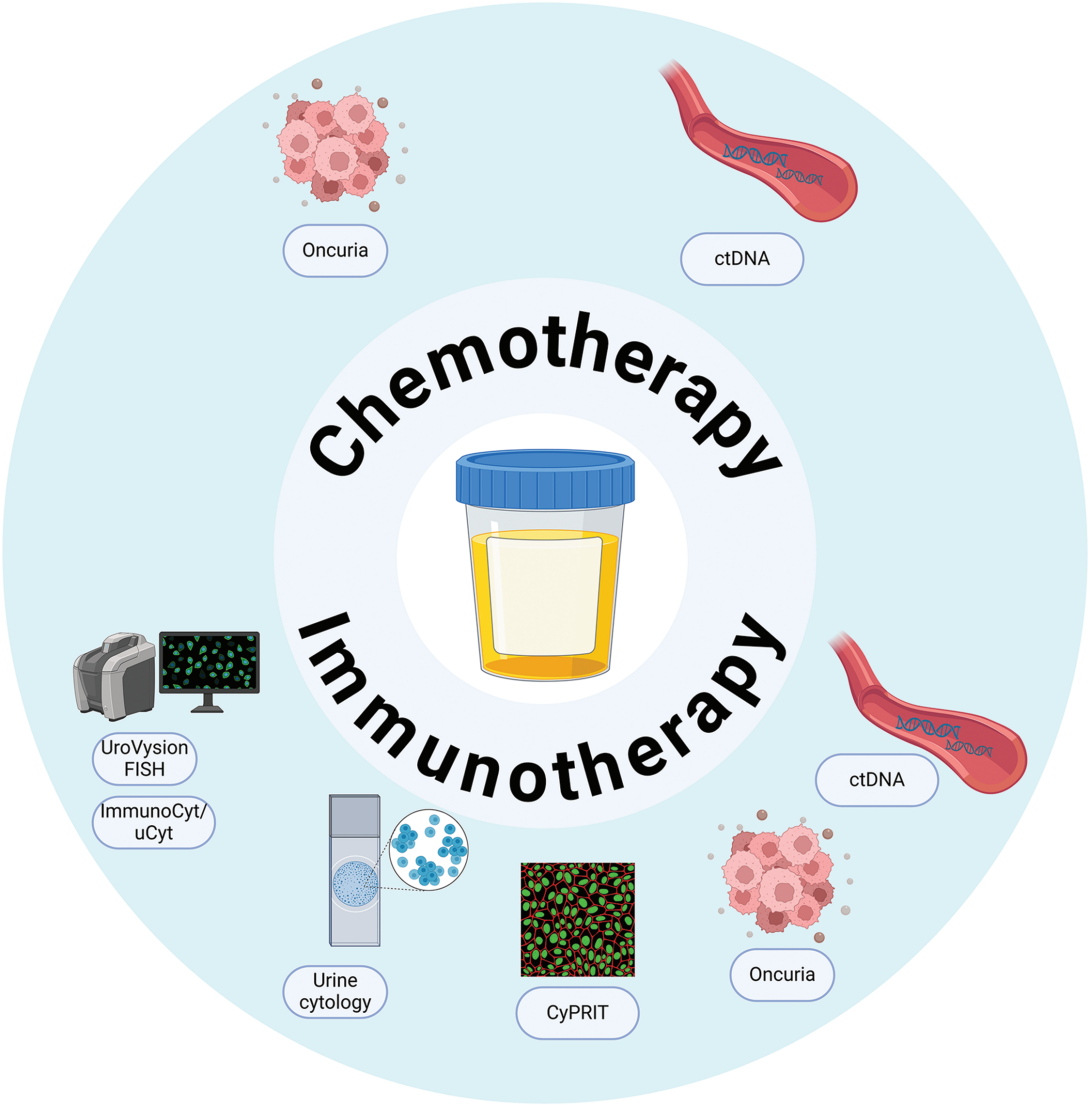
Urine biomarkers predictive of therapeutic response in bladder cancer. An overview of urine-based biomarkers—including ctDNA (circulating tumor DNA), cytology, ImmunoCyt, UroVysion FISH, Oncuria, and CyPRIT (cytokine panel for response to intravesical therapy)—that are currently being investigated for their potential to predict responses to chemotherapy and immunotherapy in bladder cancer patients.

Urine is used for biomarker evaluation due to its non-invasive nature, ease of collection, and cost-effectiveness [18]. Primarily, urine biomarkers were studied for diagnosis and monitoring purposes, for early identification of BC and improving the accuracy of diagnosis, with only a limited number of markers having the necessary accuracy to predict therapeutic response [27,28]. This section focuses on the urinary biomarker tests that provide insights into therapy response, rather than covering all known tests in the literature (Fig. 1).

According to the American Urological Association/Society of Urologic Oncology Guideline, UroVysion fluorescence *in situ* hybridization (FISH) and ImmunoCyt (both FDA-approved tests for the diagnosis of urothelial carcinoma (UC)) can be used as potential biomarkers for monitoring therapy responses, particularly in NMIBC patients undergoing BCG therapy [28]. UroVysion is a multi-chromosomal FISH assay, which is designed to detect aneuploidy in chromosomes 3, 7, and 17, as well as the loss of the 9p21 locus. For the BC diagnosis, one of these conditions is required: at least four cells with gains in at least two chromosomes within the same cell (out of 25 cells); ten or more cells showing a gain of a single chromosome; ten or more cells with tetrasomic signal patterns, or over 20% of cells showing a loss of the 9p21 locus [28]. While UroVysion FISH is not FDA-approved for predicting therapeutic outcomes, its ability to detect chromosomal anomalies in urine samples provides significant insights into the likelihood of recurrence post-therapy. In a study of 76 patients with BC who were treated with BCG and then tested using FISH, the patients who had a positive FISH test after treatment had a higher recurrence rate or progression, compared to those with a negative test. Before treatment with BCG, the recurrence rate predicted by a positive FISH test was like that predicted by a negative FISH test (10.3% *vs*. 10%). After BCG treatment, the recurrence rate for patients with a positive FISH test was significantly higher (100%) compared to those with a negative FISH test (7.1%) [29]. Moreover, based on a study published by Kamat et al., FISH testing at 6 weeks and 3 months showed that patients with positive results were more likely to experience tumor recurrence compared to those with negative results, indicating its potential to predict BCG therapy failure early on [30].

The ImmunoCyt test is an immunocytofluorescence-based assay using fluorescently labeled monoclonal antibodies, which targets carcinoembryonic antigens and sulfated mucin glycoproteins present in most BC cells. It is meant to complement urine cytology and cystoscopy, and requires a minimum of 500 cells for evaluation, considering a single fluorescent cell as indicative of a positive result [28]. ImmunoCyt is used as an adjunct to other diagnostic methods like cytology and cystoscopy, to enhance detection accuracy of UC rather than specifically predict BCG therapy outcomes. It is, however, used to manage equivocal cytology outcomes, enhancing surveillance protocols. ImmunoCyt can detect recurrent BC with a sensitivity of 73% and a negative predictive value of 80%, making it valuable for patients with atypical cytology, reducing the need for unnecessary invasive procedures and optimizing follow-up assessments [28].

The Oncuria™ urine-based assay is a validated quantitative multiplex immunoassay designed to measure the expression of cancer-associated markers in voided urine specimens. This assay helps in predicting treatment outcomes for BC by analyzing pre-treatment concentrations of various protein markers like MMP9, VEGFA, CA9, SDC1, PAI1, APOE, A1AT, ANG, and MMP10 [31]. Results published by Murakami et al. showed increased pre-treatment levels of biomarkers associated with higher recurrence rates post-BCG treatment in NMIBC patients [32]. The predictive model showed an area under the ROC curve (AUROC) of 0.89, indicating high accuracy with a sensitivity of 81.8% and a specificity of 84.9% [32]. Using Oncuria™ to assess response prediction to PD-L1 inhibitor therapy in a group of 18 NMIBC patients who did not respond to BCG and received atezolizumab, the predictive model showed high accuracy and precision (>90%) in predicting treatment results [33]. These findings could enable the selection of patients who are not likely to respond to BCG therapy and identify NMIBC patients likely to benefit from systemic immune-oncology therapies like PD-L1 inhibitors.

The post-treatment expression of certain immune markers in the urine are used as an indicator of the patient’s immune response to BCG therapy [34]. CyPRIT (cytokine panel for response to intravesical therapy) is a nomogram used to measure levels of 9 cytokines (IL-2, IL-6, IL-8, IL-18, IL-1ra, TRAIL, IFN-γ, IL-12[p70], and TNF-α) as a predictor of response to BCG treatment in intermediate- and high-risk NMIBC patients [35]. The MD Anderson Cancer Center study found that changes in the levels of these cytokines from before to just after the sixth instillation of BCG were the best predictor of recurrence, with an AUROC of 85.5% [34]. The study by Salmasi et al. showed that changes in urinary cytokine levels, specifically IL18-binding protein-a, IL23, IL8, and IFNγ-induced protein-10, could predict the time to event (recurrence or progression) in patients with NMIBC undergoing immune-modulating treatments [36]. This aligns with findings from Liu et al., which highlighted the importance of cytokines IL-2, IL-8, and TNF-α as positive biomarkers for treatment response and nonrecurrence, and IL-10 for a negative association with BCG therapy response [37]. Watanabe et al. [38] evaluated cytokine levels in 20 CIS patients following their first and eighth BCG instillation. They found that urinary IL-2 levels measured after the final instillation were independently linked to the patients’ response to treatment.

Urinary circulating tumor DNA (ctDNA) shows promise as a significant biomarker for monitoring therapy response and guiding treatment management. The elevated level of ctDNA in the urine of patients prior to RC has been correlated with a lack of response to NAC and poor outcomes following RC [39,40]. Multiple studies have shown promising results for ctDNA analysis with the potential to influence MIBC patient management [41]. At the initial diagnosis of MIBC, the absence of ctDNA is associated with a better prognosis, potentially allowing patients to avoid NAC. Patients with detectable ctDNA are at higher risk and should undergo NAC with ongoing ctDNA monitoring. If ctDNA clears during treatment, additional chemotherapy cycles may be beneficial, whereas persistent ctDNA suggests the need for a treatment change or early RC. Post-RC ctDNA detection indicates metastatic disease and could prompt treatment initiation regardless of imaging results. In metastatic cases, ctDNA monitoring can provide real-time feedback on treatment efficacy, guiding regimen changes if ctDNA persists [40,41].

In a study conducted by Hu et al. [42], the authors examined the clinical utility of urine tumor DNA (utDNA) in BC patients. They analyzed paired tDNA and utDNA samples from 48 BC cases. Results showed high consistency between tDNA and utDNA profiles, with 75.4% of tDNA mutations detected in utDNA. The mutational landscape of the top 20 genes in utDNA closely resembled that of tDNA, indicating that urine can be a viable sample for noninvasive detection and monitoring of BC. Notably, the study found that ctDNA dynamics correlated with treatment response. Responders exhibited higher consistency between tDNA and utDNA mutations compared to non-responders, with 70.1% of tDNA mutations detected in utDNA for responders *vs*. 25.0% for non-responders.

## Tissue Based Biomarkers

Non-muscle-invasive bladder cancer (NMIBC) patients are currently stratified into risk groups based on clinical and histopathological factors [43]. However, high rates of recurrence and progression, along with systemic side effects from treatments, highlight the need for better risk stratification methods. The use of biomarkers in predicting recurrence, progression, and response to BCG therapy can improve NMIBC management and may guide decisions on early cystectomy [44]. MIBC is a complex disease characterized by various molecular alterations that may serve as prognostic biomarkers. Current studies are focusing on identifying and validating certain biomarkers to improve patient stratification and develop personalized treatment approaches [27,45].

### Intravesical therapy

Several biomarkers have been evaluated as potential predictors of response to intravesical therapies. In a multicenter trial, Malmström et al. [46] assessed the predictive value of ezrin, CK20, and Ki-67 for response to BCG and intravesical chemotherapy. However, none of these biomarkers were able to predict treatment response, with only tumor multifocality being associated with disease progression [27].

De Jong et al. [47] classified BCG response into three molecular subtypes (BRS 1, 2 and 3). The BCG response subtype 3 (BRS3) showed the worst PFS and highest recurrence, and was characterized by overexpression of immunosuppressive genes, including PD-1/PD-L1 and chemokines, as well as markers of basal and epithelial-to-mesenchymal transition (EMT) activation markers. Similarly, Kim et al. [48] identified three molecular subtypes with distinct prognostic features: DP.BCG+ (associated with progression despite BCG response), REC.BCG+ (associated with recurrence despite BCG response), and EP (equivocal prognosis). The DP.BCG+ subtype showed poor PFS, while the REC.BCG+ subtype was linked to worse recurrence-free survival (RFS) despite BCG treatment response [48].

Using next-generation sequencing (NGS), Pietzak et al. [49] analyzed pretreatment tumor samples from 105 BCG-naïve patients with NMIBC. Using targeted exon sequencing, they identified genetic alterations and examined their clinical implications, particularly focusing on *TERT* promoter and *ARID1A* mutations. *TERT* promoter mutations were found in 73% of tumors, suggesting early occurrence in tumorigenesis. Chromatin-modifying gene alterations, particularly *ARID1A* mutations, were present in 69% of cases. *ARID1A* mutations were significantly associated with an increased risk of recurrence after BCG therapy, with a hazard ratio (HR) of 3.14. These mutations were linked to poorer RFS post-BCG therapy, indicating their role as predictive biomarkers.

Epigenetic changes also influence the response to BCG therapy. Agundez et al. [50] examined the methylation status of 25 tumor suppressor genes in T1G3 NMIBC patients treated with BCG and identified gene combinations predictive of disease progression. Alvarez-Múgica et al. [51] conducted a retrospective analysis of 108 NMIBC cases, finding that the absence of methylation in the *PMF-1* gene, which is involved in cellular proliferation, was linked to a higher risk of recurrence and progression.

### Systemic chemotherapy

The administration of NAC has as primary roles the tumor-size reduction and micrometastases treatment. However, only 40% of MIBC patients show a pathologic response (<pT1, N0 at RC) [52]. Therefore, delaying RC in non-responders worsens the prognosis, making it necessary to identify patients who will benefit from NAC. Various biomarkers have been studied to assess the response to cisplatin-based NAC, including those involved in the cell cycle, such as DNA repair genes, apoptosis regulators, receptor tyrosine kinases, and transmembrane transport genes, as well as molecular subtyping. The most promising biomarkers for predicting chemotherapy response are proteins related to DNA damage detection and repair (DDR) [53].

*ERCC1* and *ERCC2* are nucleotide excision repair (NER) proteins involved in the repair of damaged DNA [19]. The role of *ERCC1* in predicting NAC response is still being debated [54]. Ozcan et al. [55] found that high *ERCC1* expression can correlate with worse disease-free survival (DFS) and OS, in NAC-treated patients (a median OS of 9.3 *vs*. 26.7 months for high *vs*. low *ERCC1* expression). In contrast, Van Allen et al. identified *ERCC2* as the only gene significantly mutated in cisplatin responders compared to non-responders, based on whole-exome sequencing of 50 MIBC patients treated with NAC and RC [56]. Hirotsu et al. [57] investigated the role of genomic profiling in UC, focusing on the *ERCC2* mutation and its impact on therapy response. Using a custom panel targeting 71 genes, the study performed targeted sequencing on paired samples, tumor and blood, from 19 patients, identifying 142 somatic mutations, with 49% classified as oncogenic. Frequent mutations were observed in *KDM6A, KMT2D, TP53, KMT2C, PIK3CA*, and *ERCC2*. A remarkable finding was that a patient with an *ERCC2* helicase domain mutation showed a significant response to NAC, highlighting the potential of targeted therapy based on specific genetic alterations. The study also observed a rapid decrease in tumor-derived mutations in urine after NAC, suggesting this approach could monitor treatment response and guide therapy adjustments. Case studies demonstrated that patients with the *ERCC2* mutation responded well to carboplatin-based chemotherapy, with no recurrence observed for up to 2.5 years post-treatment [56,57].

Also, alterations in DNA repair genes *ATM, RB1*, and *FANCC*, have been associated with response to platinum-based NAC in MIBC patients. Plimack et al. [58] showed that alterations in one or more of these genes were associated with complete pathologic response.

The ERBB family of tyrosine kinase growth-factor receptors has been also analyzed for response to NAC. One study analyzed the ERBB family in 38 complete responders (ypT0N0) and 33 non-responders (>ypT2). *ERBB2 (HER2)* mutations were found in 9 responders but in none of the non-responders, suggesting that *ERBB2* mutations may predict a favorable NAC response, with HER2 expression serving as a potential marker for stratifying MIBC patients who benefit from platinum-based therapy [59].

Concerning apoptosis regulators, patients with nuclear p53 overexpression had a significantly higher rate of cancer-related deaths. Multivariable analysis confirmed that p53 overexpression was an independent predictor of survival. Long-term survival was notably lower in patients with p53 overexpression (41%) compared to those without p53 overexpression (77%) [60].

The *BRCA1* gene encodes a nuclear protein that responds to DNA damage by participating in gene transcription, DNA damage repair, cell growth, and apoptosis. Font et al. [61] evaluated *BRCA1* mRNA expression in 57 MIBC patients before NAC treatment. Their analysis of post-NAC RC specimens revealed a significant pathological response (pT0-1) in 66% of patients with low/intermediate *BRCA1* levels, compared to 22% in patients with high *BRCA1* levels. Median OS was 168 months for those with low/intermediate levels, *vs*. 34 months for those with high *BRCA1* levels. The survival analysis showed that only *BRCA1* expression levels and LVI were independent prognostic factors [61].

Cell cycle regulators, like cyclins and cyclin-dependent kinases, have been considered as potential indicators of chemotherapy response in advanced and metastatic BC. High nuclear levels of Cyclin D1 have been associated with positive outcomes in patients receiving systemic chemotherapy [27].

Genomic profiling has enhanced our understanding of BC heterogeneity and paved the way for a classification into molecular subtypes [62]. Over the past decade, several molecular classification systems have been developed, including a recent international consensus [63]. The basal and luminal subtypes are consistently recognized across major classification systems, with various studies highlighting their distinct responses to neoadjuvant therapy [64–67].

The Cancer Genome Atlas (TCGA) [62] identified four distinct subtypes of high-grade MIBC based on mRNA cluster analysis. One subtype (Cluster I) was characterized by papillary-like morphology, increased *FGFR3* expression, mutations and copy number gain, decreased miR-99a and miR-100 expressions, which in turn downregulate *FGFR3* expression, suggesting potential responsiveness to FGFR inhibitors. Two subtypes (Clusters I and II) resembled luminal A subtype of breast cancer. These two subtypes showed expressions for luminal markers like *GATA3* and *FOXA1*, uroplakins, E-cadherin and members of the miR-200 family and increased expression of *ERBB2* and estrogen receptor-β, potentially making them responsive to hormonal therapies. A third subtype (Cluster III) shared features with basal-like breast cancers and squamous cell carcinomas of the head and neck and lung, overexpressing epithelial lineage genes. Clusters III and IV contained basal or mesenchymal markers characteristic of the basal BC subtype [68].

Seiler et al. [66] noted that patients with luminal BC subtype had the best OS, regardless of NAC treatment, whereas basal-type patients showed the most significant improvement with NAC compared to surgery alone. Similarly, Lotan et al. [69], in a study of 601 MIBC patients (40% receiving NAC and 60% undergoing cystectomy alone), found that non-luminal tumors benefited the most from NAC, with a 5-year OS benefit of 10%, while luminal tumors did not exhibit a significant survival benefit. These findings suggest that NAC may be most effective for patients with non-luminal BC subtypes.

Choi et al. [70] showed that BC patients with the basal subtype, characterized by enriched gene expression in the p63 pathway, responded well to neoadjuvant MVAC, while patients with “p53-like tumors,” with activated wild-type *p53* gene expression signatures, were resistant to chemotherapy. This was further confirmed by a gene expression profiling study from a Phase 2 trial using dose-dense MVAC and bevacizumab, which indicated that basal tumors had significantly better survival rates (91%) compared to luminal (73%) and p53-like tumors (36%), which were associated with bone metastases and chemoresistance [67].

The study by Gouin et al. [71] describes a distinct subset of MIBC cells with a high expression of N-Cadherin 2 seen in both luminal and basal subtypes of BC. These *CDH12*-enriched tumors are more aggressive and resistant to chemotherapy but respond better to immunotherapy, offering a new way to personalize treatment for these patients.

While some studies have been able to show a difference in response to NAC based on molecular subtype, other studies have not found this association [72].

Although guidelines recommend NAC before RC [73,74] as it improves 5-year cancer-specific survival by 5%–10% compared to surgery alone and despite strong evidence supporting cisplatin-based NAC for MIBC, its use is limited due to concerns about delaying surgery in non-responders, the potential for toxicity, and the inability to predict which patients will benefit [52,75]. It is equally important to examine how biomarkers may guide the effectiveness of adjuvant chemotherapy in managing residual disease and improving patient outcomes.

Adjuvant therapy for MIBC involves the use of cisplatin-based chemotherapy regimens following RC to target micrometastatic disease and improve patient survival rates [76]. This treatment is particularly recommended for high-risk patients, including those with lymph node involvement or positive surgical margins. However, the overall benefit of adjuvant chemotherapy (AC) has been debated due to conflicting study results [76]. Sternberg et al. [77] indicated a clear survival benefit with multi-agent cisplatin-based chemotherapy (MVAC). They reported an improvement in 5-year OS from 50% to 56%, along with significant improvements in RFS and metastasis-free survival, compared to observation alone [77]. Similarly, the trial by Cognetti et al. [78] supported the use of AC by showing reduced rates of disease recurrence and metastasis, particularly in high-risk patients. They used a combination of cisplatin, methotrexate, vinblastine, and doxorubicin, reinforcing the role of multi-agent chemotherapy in adjuvant treatment [78]. In contrast, the trial by Freiha et al. [79] found no significant improvement in OS among patients treated with cisplatin, vinblastine, and methotrexate (CVM) compared to those undergoing surgery alone, suggesting that the modest benefits observed in other trials might not justify the added toxicity for all patients.

Mutations in DDR pathways can also enhance the effectiveness of platinum-based AC in BC patients [80]. Yap et al. [81] examined 43 patients who received AC and underwent whole-exome sequencing. They found that 25 out of 43 tumors carried mutations in at least one DDR gene. These somatic DDR gene mutations were associated with significantly improved RFS after AC—median RFS was 32.4 months compared to 14.8 months in patients without such mutations [81].

Drug transporters represent another category of biomarkers that have been studied for their role in predicting therapy response. Multidrug resistance (MDR) to chemotherapeutic drugs is a common cause of chemotherapy failure in cancer treatment. In BC, MDR can be caused by the expression of ATP-dependent efflux pumps like the drug transporter family ATP-binding cassette transporter (ABC), with its subfamily B, member 1 (ABCB1) [82]. This transporter actively expels drugs from cancer cells, reducing intracellular drug levels and diminishing the efficacy of chemotherapy [82].

MDR reversal agents function by inhibiting the drug-efflux activity of transporters like ABCB1, thus increasing intracellular drug concentrations [83]. Chemoresistance in cancer is frequently driven by the overexpression of ABCB1 in tumor cells [84,85]. Although cisplatin is not an ABCB1 substrate, studies show that prolonged gemcitabine treatment can induce ABCB1 upregulation in BC cells, contributing to drug resistance [82,86]. Therefore, ABCB1 expression may influence the efficacy of candidate drugs for NAC, or AC in BC after the failure of first-line treatment with gemcitabine and cisplatin.

MicroRNA-218 (miR-218), another transporter engaged in chemoresistance, is often downregulated in various malignancies [82]. It targets glucose transporter 1 (GLUT-1). In BC cell lines T24 and EJ, overexpression of miR-218 led to a significant reduction in glucose uptake and total glutathione levels, thereby enhancing the cells’ sensitivity to cisplatin [87].

Another example is the high-affinity copper transporter 1 (CTR1), which plays a key role in the cellular uptake of cisplatin. The expression level of CTR1 can influence the sensitivity of BC cells to cisplatin treatment. Kilari et al. [88] analyzed the expression of CTR1 in 47 MIBC patients treated with NAC. The study showed that CTR1 expression correlates with pathological outcomes, indicating that CTR1 may be predictive of cisplatin efficacy.

As for determining responses to AC regarding the BC molecular subtypes, the study by Olah et al. [89] analysed 191 patients who underwent RC with or without AC. Using a 48-gene panel, molecular subtypes were classified into luminal or basal, following TCGA, MDA, LundTax, and Consensus classification systems [62,63,65,90]. The study found that patients with luminal subtypes, particularly luminal papillary and urothelial-like tumors, showed significantly better OS when treated with AC. In contrast, basal subtypes did not show a survival benefit from AC, indicating that these patients may not derive the same advantage from platinum-based chemotherapy [89]. In addition to subtype classification, the study examined 12 single genes that might influence chemotherapy response. Among these, *APOBEC3G* emerged as a significant predictor of improved outcomes in chemotherapy-treated patients, showing better OS and higher pathological complete response rates compared to patients with low *APOBEC3G* expression [89].

### Immunotherapy

PD-L1 expression is the most studied biomarker for predicting response to ICIs. High PD-L1 expression on tumor cells or tumor-infiltrating immune cells generally correlates with better response rates to ICIs, as seen in several clinical trials. However, the inconsistency in assay methodologies and cutoff values for PD-L1 expression poses a challenge to its reliability and reproducibility across different studies and clinical settings.

The identification of PD-L1 on tumor specimens through immunohistochemistry (IHC) has been utilized in numerous clinical trials to assess the potential of PD-L1 expression as a predictive biomarker. The assessment of PD-L1 encounters various challenges due to the lack of standardization. In the IMvigor 210 trial, patients who had previously received cisplatin treatment (cohort 2) showed better objective response rates (ORR) to atezolizumab when they had higher levels of PD-L1 expression. Specifically, those with ≥5% PD-L1-positive immune cells had a 27% ORR, while those with ≥1% had an 18% response rate [91]. However, in the cisplatin-ineligible group, the ORR was not influenced by PD-L1 status [92]. On the other hand, the CheckMate 032 trial found no significant difference in ORR between patients with PD-L1 expression <1% and those with PD-L1 expression ≥1% (26.2% *vs*. 24.0%, respectively) [93]. In CheckMate 275, which assessed nivolumab in metastatic urothelial carcinoma (mUC) post-platinum therapy, the association was confirmed as ORRs were better for patients with higher PD-L1 expression (ORR of 28.4%, 23.8%, and 16.1% in patients with PD-L1 expression of ≥5%, ≥1%, and <1%, respectively) [94]. Finally, in the KEYNOTE-045 trial, the response to pembrolizumab seemed to be independent of PD-L1 expression, either on tumor cells or infiltrating immune cells [95].

Further analysis of the cisplatin-pretreated group in the Imvigor 210 trial, indicated that TCGA subtypes were independently predictive of response to atezolizumab therapy [91]. While atezolizumab response was observed across all TCGA subtypes, the luminal Cluster II subtype showed the highest response rate [91]. Similarly, in cisplatin-ineligible patients, responses were observed across all subtypes, with the highest response rate found in the luminal II subtype [92]. In contrast, findings from the CheckMate 275 trial indicated that the basal 1 subtype had the greatest proportion of responders [94].

Mutations in DDR genes, such as *ATM, BRCA1/2*, and *ERCC2*, are associated with increased sensitivity to ICIs. These mutations lead to genomic instability and higher neoantigen loads, enhancing the immune systems’ ability to recognize and target cancer cells [68]. In mUC, DDR gene alterations are associated with improved clinical outcomes in patients treated with ICIs. A study of 60 mUC patients revealed that those with DDR alterations, especially deleterious mutations, had higher response rates to ICIs compared to those with wild-type DDR genes [53,96].

Regarding ctDNA, the IMvigor010 trial demonstrated improved DFS in patients with detectable ctDNA who received adjuvant atezolizumab, compared to those under observation alone after RC, while no survival benefit was seen in ctDNA-negative patients who underwent RC [97]. This indicates that adjuvant immunotherapy might be best reserved for ctDNA-positive patients, potentially reducing unnecessary ICI toxicity [68]. Additionally, ctDNA may serve as a dynamic tool for monitoring treatment response, although further validation in randomized trials is needed.

Tumor Mutational Burden (TMB) is being investigated as a potential biomarker for immunotherapy response in BC. TMB is defined as the number of somatic mutations per megabase detected by NGS, excluding single nucleotide polymorphisms, germline mutations, copy number variations, and structural variations [98]. A high TMB is associated with an increase of tumor-specific neoantigens, which enhances the immune system to recognize and target cancer cells, thus improving treatment response. While TMB cutoffs for defining “high” vary (Foundation Medicine uses 20 mutations per megabase (mut/Mb) for high TMB [99], while the FDA uses 10 mut/Mb [100]), studies in BC have shown promising results, with high TMB often correlating with improved response rates and prognosis following ICI treatment. However, not all clinical trials have demonstrated a clear survival benefit with ICIs in high TMB BC patients. The IMvigor211 trial with atezolizumab [98] and the DANUBE study with durvalumab [101] did not show significant OS improvement in the high TMB cohort, while the CheckMate 275 study with nivolumab [102] and the Javelin Bladder 100 study with avelumab [103] did demonstrate improved outcomes in patients with high TMB. These mixed results highlight the need for further research to clarify the role of TMB as a predictive biomarker for ICI therapy in BC.

Microsatellite instability (MSI) is a distinct molecular alteration and hyper-mutable phenotype. It results from a defective DNA mismatch repair (MMR) system and can be defined as the presence of alternate-sized repetitive DNA sequences that are not present in the corresponding germline DNA [104]. MSI-high status is considered to have a better response to ICIs due to the increased production of neoantigens [68]. In a prospective study of 424 UC cases, 13 patients had high-MSI scores, with a significantly higher median mutation count compared to non-MSI patients (8 patients) [105]. On the other hand, two patients with low MSI scores but extremely high TMB presented *POLE* mutations. In upper tract UC, MSI was found in a small percentage of patients, with many having Lynch syndrome or *MSH2* mutations, and some showing high response rates to ICI [105]. The same paper showed that five mUC patients with high-MSI receiving ICI therapy, achieved near-complete or complete responses [105]. While MSI is a key biomarker in colorectal and endometrial cancers, its role in BC is still being explored, with preliminary data suggesting its potential predictive value for ICI therapy.

The tumor immune microenvironment (TIME) component, particularly tumor-infiltrating lymphocytes (TILs), has gained attention due to the introduction of ICIs. Studies show that CD8 lymphocytes in TIME have antitumor roles, especially in BC [68]. A small study found that the ratio of CD8^+^ T cells to regulatory T cells (Tregs) is linked to better responses to NAC and the high TIL density correlates with increased PD-L1 IHC expression (>5%) [106]. In patients with mUC treated with nivolumab, high CD8^+^ T cell infiltration led to better clinical outcomes [94]. Similar results were seen in the IMvigor210 trial with atezolizumab [107]. A meta-analysis of 33 studies supported these findings, suggesting high CD8^+^ TILs correlate with improved outcomes in ICI-treated cancer patients, regardless of treatment type, cancer type, or CD8^+^ T-cell location [108].

The interaction between TIME and cancer cells shapes the anticancer immune response, however systemic inflammation can disrupt the tumor inflammatory environment, and can lead to immune resistance in cancer patients. Several prognostic scores using peripheral inflammatory parameters have been developed for genitourinary cancers, including the Lung Immune Prognostic Index (LIPI) and the Bellmunt score, which have demonstrated moderate predictive accuracy [68]. The systemic immune-inflammation index, another scoring system, is defined by the neutrophil*platelet/lymphocyte ratio, and shows a higher predictive value for poor outcomes in BC compared to traditional ratios like platelet-to-lymphocyte or neutrophil-to-lymphocyte ratios [109]. These inflammatory scores consistently identify systemic inflammation as a negative prognostic factor in mUC [68]. Several meta-analyses confirm the prognostic value of pretreatment neutrophil-to-lymphocyte ratio (NLR) and lactate dehydrogenase (LDH) levels in mUC [110–112]. Additionally, C-reactive protein (CRP) has been validated as a surrogate marker for local and systemic inflammation. In a prospective study of 154 patients, CRP dynamics correlated with ICI response [113]. CRP flare responders and CRP responders showed significantly better objective response rates and prolonged PFS and OS compared to CRP non-responders. However, these scores, while assessed in both chemotherapy and ICI treated groups, are currently limited to predicting patient prognosis and not treatment response.

### Radiotherapy

**Figure 2:**
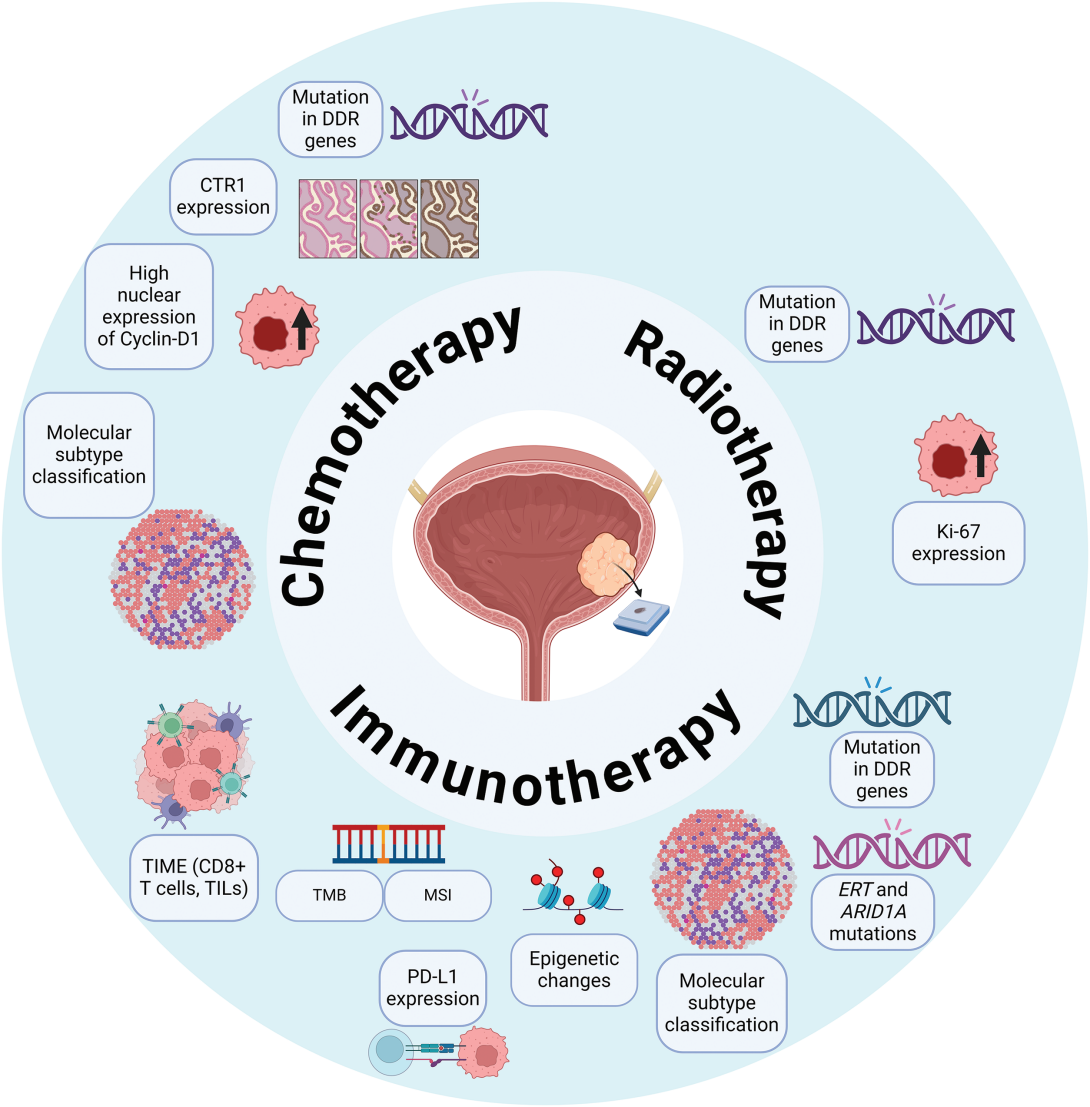
Tissue-based predictive biomarkers for therapeutic response in bladder cancer. An overview of tissue-based biomarkers that have been explored for their potential in predicting responses to chemotherapy, radiotherapy, and immunotherapy in bladder cancer patients. Abbreviations: DDR—DNA Damage Repair; CTR1—Copper Transporter Receptor 1; TIME—Tumor Immune Microenvironment; TMB—Tumor Mutational Burden; MSI—Microsatellite Instability; PD-L1—Programmed Death-Ligand 1.

Patients who are considered unfit for RC may benefit from radiotherapeutic treatment as part of trimodal therapy (TMT). Rödel et al. [114] showed a correlation between a high Ki-67 index (≥8%) and complete response as well as local control, resulting in the preservation of the bladder five years post-radiotherapy. In a retrospective analysis, Tanabe et al. [115] found that high Ki-67 expression (≥20%) was independently associated with improved cancer-specific survival. Patients with high Ki-67 levels had a 5-year survival rate of 78%, compared to 46% for those with lower Ki-67 expression. Alternatively, Matsumoto et al. [116] did not find any association between the expression of p53, Bcl-2, and Bax, and radiotherapy response. The existence of DDR gene alterations, detected in *ERCC2*, showed a positive response to radiotherapy, resulting in improved RFS after a two-year monitoring period [117] (Fig. 2).

## Serum Biomarkers

**Figure 3:**
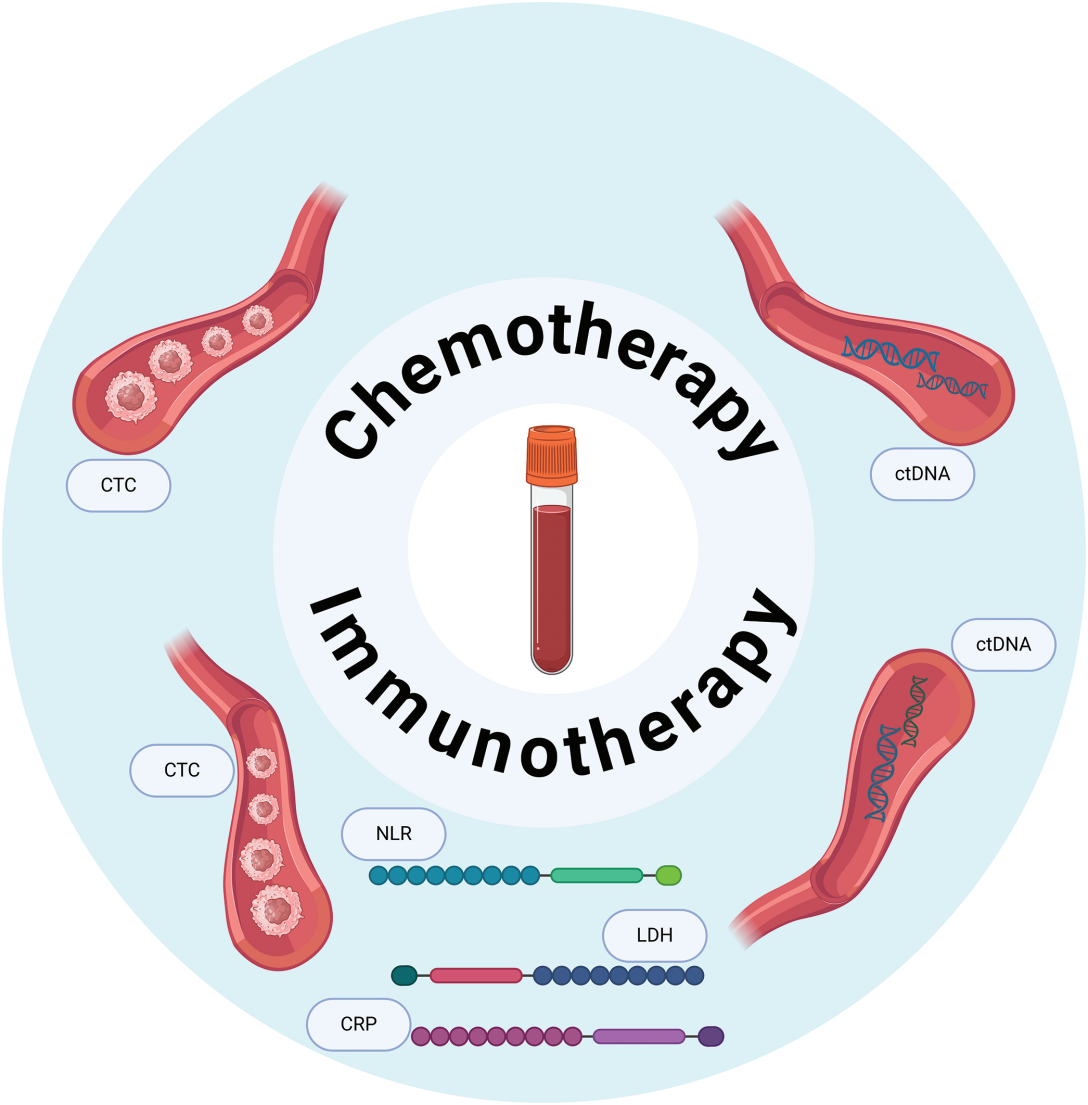
Blood-based biomarkers predictive of therapeutic response in bladder cancer. An overview of blood-based biomarkers investigated for their potential to predict responses to chemotherapy and immunotherapy in bladder cancer patients. These biomarkers include circulating tumor cells (CTCs), circulating tumor DNA (ctDNA), and other markers such as neutrophil-to-lymphocyte ratio (NLR), C-reactive protein (CRP), and lactate dehydrogenase (LDH).

Serum biomarkers for BC are a growing area of research (Fig. 3). The liquid biopsy tests offer the potential for assessing cancer risk stratification, molecular profiling, predicting treatment response, and tumor surveillance [18]. While primarily used in preclinical studies thus far, these tests are gaining clinical relevance in guiding treatment decisions.

Circulating tumor cells (CTCs) were among the first serum biomarkers to be studied. CTCs are malignant cells in peripheral blood originating from the primary tumor [19]. Despite a sensitivity of only 35% in detecting BC, attributed to the limited presence in the bloodstream, the detection of CTCs has been associated with high-grade, lymph node involvement, and the presence of metastasis [118]. Moreover, studies found that CTCs were detectable in 20%–30% of NMIBC patients and in 40%–100% of MIBC patients, with higher counts in high-grade BC [118,119]. Yang et al. [120] measured pre-treatment CTCs in 32 NMIBC patients undergoing NAC followed by RC. The tetraploid CTCs effectively identified NAC-sensitive patients from NAC-resistant ones, with an AUC of 0.80.

Similarly, extracellular vesicles (EVs) have attracted interest for their diagnostic and prognostic potential in BC. EVs are lipid-bound particles released by cells that contain nucleic acids, proteins, and metabolites, and contribute to intercellular communication. EVs are classified into exosomes, microvesicles, and apoptotic bodies based on their biogenesis. They are considered promising tools for diagnosis and prognosis in liquid biopsies, including urine, plasma, and serum [121]. EVs, containing genetic information and proteins, are released by nearly all cell types, including tumor cells. Tumor EVs have been shown to promote cancer progression in multiple cancers, including BC [19]. One study reported significantly higher concentrations of EVs in BC patients compared to healthy controls, with a sensitivity of 81% and specificity of 90% [122].

miRNAs such as miR-375, miR-146a, and miR-21-5p in urinary EVs have been identified as potential diagnostic biomarkers for different grades of BC. miR-375 serves as a diagnostic marker for high-grade BC, miR-146a for low-grade BC and miR-21-5p is particularly useful in detecting BC in patients with negative urine cytology results, with a sensitivity of 75.0% and specificity of 95.8% [121]. Similarly, lncRNAs such as MALAT1, PCAT-1, and SPRY4-IT1 in EVs have shown superior diagnostic performance compared to urine cytology, and some lncRNAs have been associated with poor RFS in NMIBC patients [121]. Zhan et al. [123] examined the expression of eight lncRNAs in urinary EVs and discovered that increased expression of PCAT-1 and MALAT was associated with poor RFS. Proteomic analysis of urinary extracellular vesicles (uEVs) has identified proteins, such as TACSTD2, HSP90, syndecan-1, and MARCKS, that are enriched in BC patients.

CtDNA serves as a minimally invasive biomarker with significant potential for monitoring treatment response and early detection of metastatic disease in MIBC. Plasma ctDNA can be collected from a simple blood draw, avoiding the need for invasive surgeries to obtain tumor samples, making it easier and safer for patients. The study by Lindskrog et al. [124] analyzed serum ctDNA in patients before and after RC, providing a median follow-up of 72 months. The detection of ctDNA post-RC demonstrated a high sensitivity (94%) and specificity (98%) for identifying metastatic relapse. The research also found that ctDNA dynamics during NAC were independently associated with patient outcomes. Vandekerkhove et al. [125] analyzed plasma ctDNA from 104 metastatic BC patients and compared them to the same-patient tumor tissue genetic profile. The study found an 83.4% match between mutations in ctDNA and those in tumor tissue, suggesting that ctDNA is a reliable substitute for tissue samples in identifying genetic mutations. High ctDNA levels were found to be independently prognostic for OS in patients initiating first-line systemic therapy. Additionally, the levels of ctDNA in the blood subsided after treatment, indicating that ctDNA can be used for therapy response.

All in all, CTCs and EVs could help improve the detection, risk assessment, and monitoring of BC patients. Identifying specific miRNAs, lncRNAs, and proteins within EVs may help predict patient responses to therapies. However, further validation through larger prospective clinical trials is needed to confirm their clinical utility.

## Conclusions

In conclusion, the identification and validation of biomarkers for predicting BC therapy response represent a crucial advancement in personalized medicine. The heterogeneity of BC necessitates a multifaceted approach to treatment, and reliable biomarkers can significantly enhance therapeutic outcomes by enabling tailored treatment strategies. Current research has identified several promising biomarkers, including genetic, epigenetic, proteomic, and transcriptomic markers, which show potential in predicting responses to chemotherapy, immunotherapy, and targeted therapies.

Despite these advancements, several challenges remain. The variability in biomarker performance across different patient populations, the need for large-scale validation studies, and the integration of biomarker testing into clinical practice are critical issues that must be addressed. Additionally, the development of robust, reproducible assays and the standardization of biomarker assessment methodologies are essential to ensure consistent and reliable results.

Future research should focus on longitudinal studies to evaluate the predictive power of these biomarkers over time, as well as on the exploration of combination biomarker panels to improve predictive accuracy. Furthermore, advancements in bioinformatics and machine learning offer promising avenues for the integration and interpretation of complex biomarker data, potentially leading to more precise and individualized treatment regimens.

Ultimately, the successful incorporation of predictive biomarkers into clinical practice has the potential to revolutionize BC therapy, improving patient outcomes and reducing the burden of unnecessary treatments. Continued collaboration between researchers, clinicians, and regulatory agencies will be vital to overcoming current barriers and fully realizing the potential of biomarkers in BC care.

## Data Availability

Data sharing not applicable to this article as no datasets were generated or analyzed during the current study.
